# Association of Plasma Glucose to Potassium Ratio and Mortality After Aneurysmal Subarachnoid Hemorrhage

**DOI:** 10.3389/fneur.2021.661689

**Published:** 2021-05-04

**Authors:** Hyun Min Jung, Jin Hui Paik, Sin Young Kim, Dae Young Hong

**Affiliations:** ^1^Department of Emergency Medicine, Inha University School of Medicine, Incheon, South Korea; ^2^Department of Emergency Medicine, Konkuk University School of Medicine, Seoul, South Korea

**Keywords:** aneurysmal subarachnoid hemorrhage, biomarkers, glucose, potassium, prognosis

## Abstract

**Objectives:** Hyperglycemia and hypokalemia are common problems in patients with aneurysmal subarachnoid hemorrhage (aSAH). The aim of this study was to determine whether the plasma glucose to potassium ratio (GPR) predicts mortality due to aSAH.

**Methods:** We prospectively recruited aSAH patients and healthy controls between March 2007 and May 2017. Clinical outcomes included mortality and poor outcome (modified Rankin scale score of 3-6) after 3 months. Multivariable analysis was used to determine the association between plasma GPR and 3-month mortality in aSAH patients.

**Results:** A total of 553 patients were recruited, and the mortality rate was 11%. The GPR was significantly elevated in aSAH patients compared with controls, in patients with a poor outcome than with a good outcome and in non-survivals than in survivals. Multivariable analysis showed that the plasma GPR was an independent factor associated with 3-month mortality. The area under the curve of the GPR was 0.747 in predicting 3-month mortality.

**Conclusion:** The plasma GPR on admission has potential as a predictor of 3-month mortality in patients with aSAH.

## Introduction

Aneurysmal subarachnoid hemorrhage (aSAH) has a poor prognosis despite significant advances in early diagnostic modalities and treatment (1). aSAH counts for ~5% of all acute stroke cases and has a high mortality rate of 32–44% (2). Assessment of the severity of aSAH and predicting patients' prognosis are required for the optimal treatment of aSAH. Multiple clinically based risk prediction scales are currently used to classify the severity of aSAH; the Hunt and Hess (HH) and the World Federation of Neurological Surgeons (WFNS) scale are commonly used scales for predicting the prognosis of aSAH.

Biomarkers may help clinicians to make therapeutic decisions when considering the severity of aSAH and in being able to predict whether a patient will have a poor prognosis, which may improve outcomes. Numerous biomarkers such as D-dimer, lactate, and C-reactive protein (CRP) have been reported to be the most promising biomarkers in aSAH patients (1, 3, 4). In addition, some clinical studies have reported that hyperglycemia is related with the risk of poor outcome and delayed cerebral ischemia (DCI) after aSAH (5, 6). Recent reports have suggested that the glucose to potassium ratio (GPR) is significantly correlated with cerebral vasospasm and functional outcome (7, 8). The purpose of the present study was to calculate the plasma GPR in patients with aSAH and to determine whether this ratio is associated with the risk of 3-month mortality.

## Materials and Methods

### Study Population

This was observational study undertaken from March 2007 to May 2017 in the emergency department (ED) at tertiary hospital, an 835-bed institution in Seoul with ~58,000 ED visits annually.

**Figure 1:**
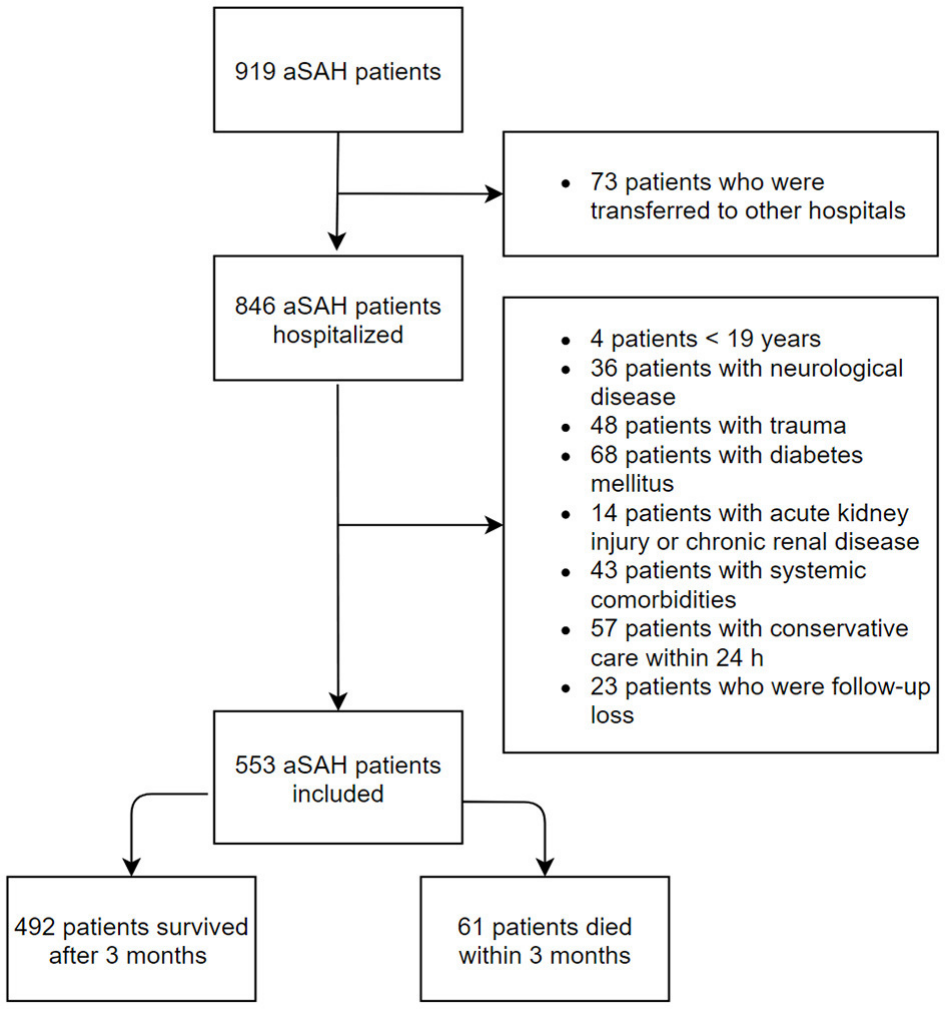
Study population flow chart.

The inclusion criteria were age ≥ 19 years and non-traumatic aSAH patients who were admitted to the ED within 24 h of symptom onset. Age- and sex-matched healthy individuals who visited our medical center for a medical examination were enrolled as the control group. The control group were checked for the absence of acute or chronic illness *via* a health questionnaire and a medical examination. The exclusion criteria were age of <19 years, a history of neurological disease including hemorrhagic or ischemic stroke and trauma, diabetes mellitus, acute kidney injury or chronic kidney disease, and concurrent systemic comorbidities including malignancy and liver cirrhosis (Figure 1).

Treatment for enrolled patients was performed according to protocols from the Korean Society of Cerebrovascular Surgeons, which included prevention of rebleeding and the management of vasospasm, delayed cerebral ischemia, seizure, and other medical complications (9). Treatment modalities included endovascular coiling or surgical clipping, as judged by both experienced endovascular specialists and neurosurgeons, which were determined based on the patients- and facility-related factors. Plasma glucose levels were checked four times per day. When the glucose level was persistently ≥ 200 mg/dl, the patients was managed using a sliding-scale insulin protocol.

The protocol of this study was approved by the Institutional Review Board of our hospital, and individual informed consent was waived owing to the routinely measured laboratory data during the process of the diagnosis and treatment in the ED.

### Data Collection and Endpoints

Peripheral venous blood samples were collected within 30 min of admission. The plasma glucose and potassium levels were measured using a TBA-200FR Neo (Toshiba Medical Systems, Tokyo, Japan). There was no equipment replacement during the study period. The reference range of the plasma glucose level in our institutions was 70–99 mg/dl, and the reference range of the plasma potassium level was 3.5–5.5 mmol/l. The patients with hypokalemia were classified as mild hypokalemia (3.1–3.4 mmol/l), moderate hypokalemia (2.5–3.0 mmol/l), and sever hypokalemia (<2.5 mmol/l).

Patients demographic characteristics, clinical features, and laboratory data were collected. Clinical severity of aSAH on ED admission was evaluated according to the HH scale. We defined severe aSAH as HH scale score 4–5. The primary endpoint of interest was death within 3 months. The secondary endpoint was poor functional outcome at 3 months following ED admission; poor functional outcome was assessed using the modified Rankin scale (mRS score of 3–6). To assess the outcomes after 3 months, a review of the medical records or a telephone interview were conducted by a physician who was blinded to the clinical information.

### Statistical Analysis

IBM SPSS Statistics 25 (IBM, Armonk, NY, USA), R version 3.6.3 (The R foundation, Vienna, Austria), and MedCalc version 16.4.3 (MedCalc Software, Ostend, Belgium) were used for all statistical analysis. Categorical variables were expressed as numbers and percentages, and proportions were compared with the χ^2^ test. Non-normally distributed continuous variables were expressed as median and interquartile range (IQRs) and were analyzed using the Mann–Whitney *U*-test and Kruskal–Wallis test for intergroup differences. The Spearman's Coefficient test was performed to assess the relationships between GPR and white blood cell (WBC) count, HH score, Glasgow Coma Scale (GCS) score, and time from symptom onset to ED admission.

The aSAH patients were divided into four groups according to their plasma GPR quartile, and overall survival was evaluated until 3 months using the Kaplan–Meier survival curves. A receiver-operating characteristics (ROC) curve was applied to identify an optimal cutoff of plasma GPR for predicting 3-month mortality, and the area under the curve (AUC) and 95% confidence interval (CI) were reported.

A multivariable regression analysis was carried out to identify independent prognostic variables for 3-month mortality in patients with aSAH. All statistical testing was two-sided, and *p* < 0.05 was considered statistically significant mortality.

## Results

### Baseline Characteristics

During the study period, 553 aSAH patients were admitted to the study in accordance with the inclusion criteria. Simultaneously, 553 age- and sex-matched control were recruited. The median (IQR) plasma glucose levels were remarkably increased in patients with aSAH compared with controls: 146 (126–172) vs. 96 (88–105) mg/dl, respectively (*p* < 0.001). Plasma potassium concentrations were lower in aSAH patients than in healthy controls: 3.7 (3.4–4.0) vs. 4.2 (3.9–4.5) mmol/l, respectively (*p* < 0.001). The plasma GPR at ED admission was significantly higher in patients with aSAH than controls: 38.9 (32.8–47.2) vs. 22.8 (20.5–26.3) (*p* < 0.001).

The demographic characteristics, clinical features, and laboratory data of enrolled subjects are summarized in Table 1. The median age was 54 (46–63) years, there were 235 (42.5%) males and 318 (57.5%) females. The median time from symptom onset to ED admission was 120 (49–280) min for all patients. The median time from symptom onset to ED admission was significantly shorter in the non-survivors than in survivors [60 (30–197) vs. 120 (56–286) min, respectively; *p* = 0.048]. Among all aSAH patients, the severe group (HH score 4–5) accounted for 24.2%. A total of 285 (51.5%) patients underwent endovascular coiling for the aneurysm, 237 (42.9%) patients underwent neurosurgical clipping, and 170 (30.7%) patients were transfused with packed red blood cells.

**Table 1:** Characteristics and outcomes of the study population.

**Characteristics**	***N* = 553**
Age (years)	54 (46–63)
Female (no.)	318 (57.5)
Time from onset to ED admission (min)	120 (49–280)
Hunt and Hess score	
Non-severe (1–3)	419 (75.8)
Severe (4–5)	134 (24.2)
Modified Fisher scoreGCS score	3 (3–4) 14 (12–15) 146 (125–171)
Laboratory results	
WBC (× 10^3^/μl)	10.6 (8.2–14.3)
Glucose (mg/dl)	146 (125–171)
Hypoglycemia (<70)	0 (0)
Normoglycemia (70–99)	9 (1.6)
Hyperglycemia (>99)	544 (98.4)
Potassium (mmol/l)	3.7 (3.4–4.0)
Hypokalemia (<3.5)	144 (26.0)
Normokalemia (3.5–5.5)	406 (73.4)
Hyperkalemia (>5.5)	3 (0.5)
Glucose/potassium ratio	38.7 (32.6–46.6)
Hemoglobin (g/dl)	13.5 (12.7–14.7)
PLT (× 10^3^/μl)	231.0 (194.5–271.0)
CRP (mg/dl)	0.09 (0.04–0.24)
Treatment modality (no.)	
Endovascular coiling	285 (51.5)
Neurosurgical clipping	237 (42.9)
RBC transfusion	170 (30.7)
Functional outcome	
Good (mRS 0–2)	399 (72.2)
Poor (mRS 3–6)	154 (27.8)
3-Month mortality rate	61 (11.0)

*Data are presented as median with interquartile ranges or number (%)*.

*GCS, Glasgow Coma Scale; WBC, white blood cells; PLT, platelet count; CRP, C-reactive protein; RBC, red blood cell; mRS, modified Rankin scale*.

A total of 544 (98.4%) patients had an elevated plasma glucose level (>99 mg/dl) on ED admission. Hypokalemia was present in 144 (26.0%) patients, but hyperkalemia was detected in only three (0.5%) patients. A lower percentage of patients suffered moderate hypokalemia (3.7%) than mild hypokalemia (22.8%), and none of patients fell into the severe hypokalemia. The plasma glucose and GPR were significantly higher in the severe group than they were in the non-severe group (180 vs. 138 mg/dl, *p* < 0.001, and 50.3 vs. 36.2, *p* < 0.001, respectively). The potassium concentration was significantly higher in the non-severe group than it was in the severe group (3.8 vs. 3.5 mmol/l, *p* < 0.001).

### 3-Month Functional Outcomes and Mortality

All enrolled subjects were categorized into four groups according to their plasma GPR quartile (Q): (1) Q1 (<32.7), (2) Q2 (32.7–38.8), (3) Q3 (38.9–47.2), and (4) Q4 (>47.2). The patients' characteristics and clinical features are presented according to GPR quartile in Table 2. Severity of aSAH, poor functional outcome, and 3-month mortality were significantly higher in the Q4 group than in the Q1 group.

**Table 2:** Clinical features according to GPR quartiles.

	**Q1 (*n* = 138)**	**Q2 (*n* = 138)**	**Q3 (*n* = 136)**	**Q4 (*n* = 141)**
Age (years)	51 (43–61)	56 (47–64)	56 (46–63)	55 (46–66)
Female (no.)	75 (54.3%)	84 (60.9%)	78 (57.4%)	81 (57.4%)
Hunt and Hess scale	2 (1,2)^b,c,d^	2 (2)^a,c,d^	2 (2,3)^a,b,d^	3 (2,4)^a,b,c^
Modified Fisher scale	2 (1,3)^b,c,d^	3 (2,3)^a,d^	3 (3)^a,d^	3 (3,4)^a,b,c^
GCS score	15 (15)^b,c,d^	15 (14,15)^a,d^	15 (13,15)^a^	13 (9,15)^a,b^
Poor outcome (no.)	20 (14.5%)^c,d^	32 (23.2%)^d^	43 (31.6%)^a^	58 (41.1%)^a,b^
3-Month mortality (no.)	2 (1.4%)^c,d^	8 (5.8%)^c,d^	22 (16.2%)^a,b^	29 (20.6%)^a,b^

*Data are presented as median with interquartile ranges or number (%)*.

*GPR, glucose to potassium ratio; Q, quartile; GCS, Glasgow Coma Scale*.

a *p < 0.05, vs. Q1*.

b *p < 0.05, vs. Q2*.

c *p < 0.05, vs. Q3*.

d *p < 0.05, vs. Q4*.

**Figure 2:**
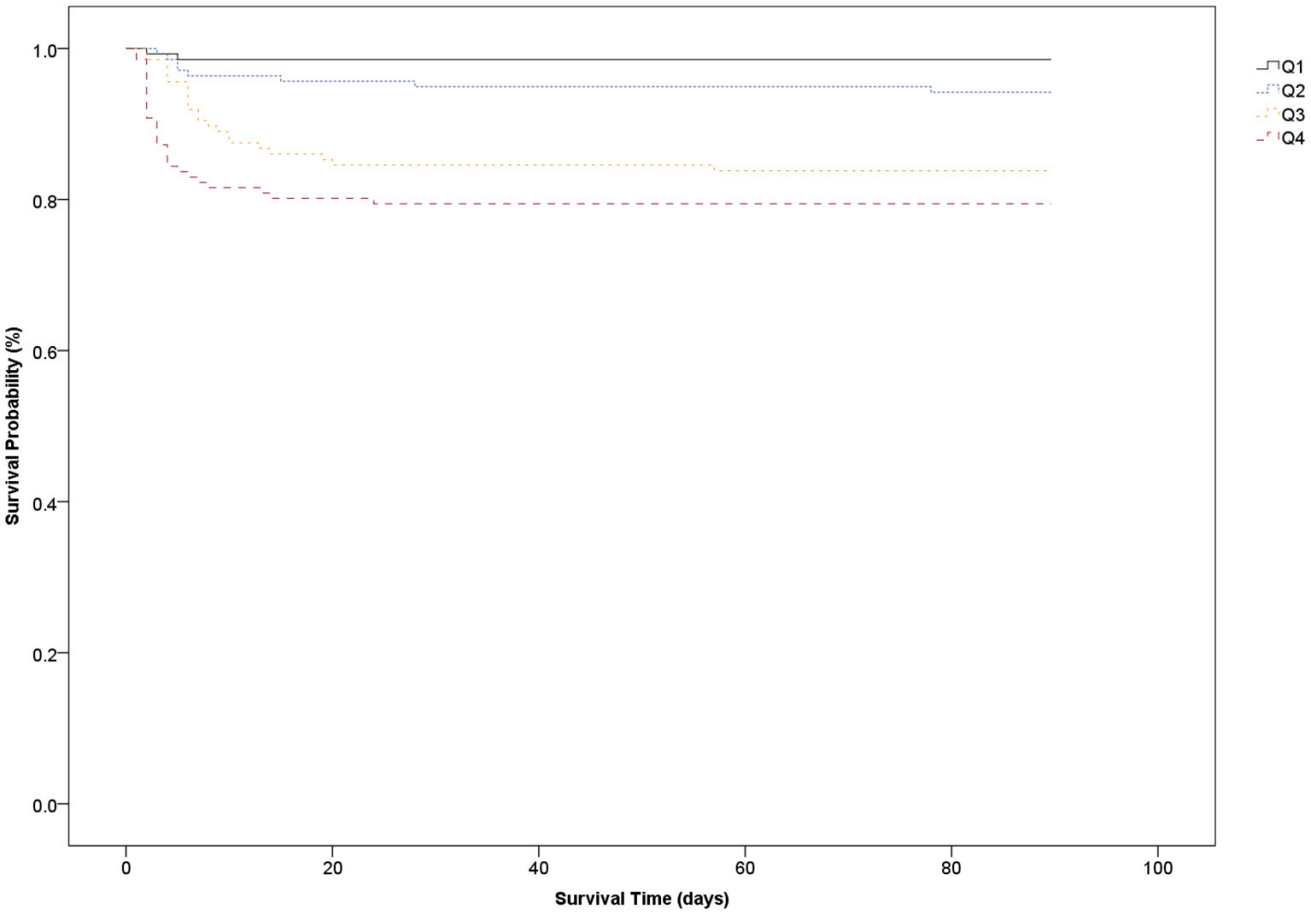
The Kaplan–Meier survival analysis based on the glucose/potassium ratio.

The survival analysis showed that the higher GPR quartile group (Q4) at ED admission had the worst 3-month survival rate whereas the lower GPR quartile group (Q1) had the best 3-month survival rate. The 3-month mortality in Q4 (GPR > 47.2) was 20.6% whereas the 3-month mortality in Q1 (GPR <32.7) was 1.4% (Figure 2).

The Spearman rank correlation test of the relationships between plasma GPR, HH score, GCS score, and WBC count produced correlation coefficients of 0.383 (GPR and HH score), −0.339 (GPR and GCS score), 0.250 (GPR and WBC count), and −0.320 (GPR and time from symptom onset to ED admission) (all *p* < 0.001). The plasma potassium level and glucose level at ED admission produced a correlation coefficient of −0.303 (*p* < 0.001).

Sixty-one (11.0%) patients died, and 154 (27.8%) patients had poor functional outcome. The median time from ED admission to death was 4 (IQR 2–8, range 2–78) days. The initial median glucose concentration, potassium concentration, GPR, and other biomarkers are presented in Table 3.

**Table 3:** Comparison of glucose and potassium levels and other biomarkers according to outcome.

	**Functional outcome**			**Survival**		
	**Good**	**Poor**	***P*-value**	**Survivors**	**Non-survivors**	***P*-value**
WBC (× 10^3^/μl)	10.0 (8.1–13.5)	11.6 (8.7–16.1)	0.058	10.2 (8.1–13.4)	14.0 (10.5–19.4)	<0.001
Hemoglobin (g/dl)	13.6 (12.8–14.6)	13.2 (11.9–14.1)	0.066	13.5 (12.8–14.5)	12.4 (11.3–16.2)	0.048
CRP (mg/dl)	0.07 (0.04–0.16)	0.15 (0.05–0.25)	<0.001	0.08 (0.04–0.21)	0.08 (0.05–0.23)	0.105
D-dimer (μg/ml)	0.99 (0.55–2.30)	1.16 (0.61–2.69)	0.366	1.13 (0.57–2.51)	0.65 (0.35–1.22)	0.034
Glucose (mg/dl)	142 (121–165)	158 (137–194)	<0.001	142 (122–167)	182 (151–219)	<0.001
Potassium (mmol/l)	3.7 (3.5–4.0)	3.7 (3.3–4.0)	0.028	3.7 (3.4–4.0)	3.6 (3.2–4.0)	0.087
Glucose/potassium ratio	37.5 (31.4–45.8)	42.8 (36.7–54.1)	<0.001	37.8 (31.8–46.2)	46.7 (41.3–61.1)	<0.001

*Data are presented as median with interquartile ranges*.

The plasma glucose concentration and GPR were significantly increased in the poor outcome group compared with the good outcome group. The median glucose concentration and GPR were significantly increased in the non-survival patients than in the survival patients (*p* < 0.001). The potassium concentration differed significantly between the good outcome group and poor outcome group (*p* = 0.028); however, the potassium concentration did not significantly differ between the non-survivors and survivors (*p* = 0.087).

**Figure 3:**
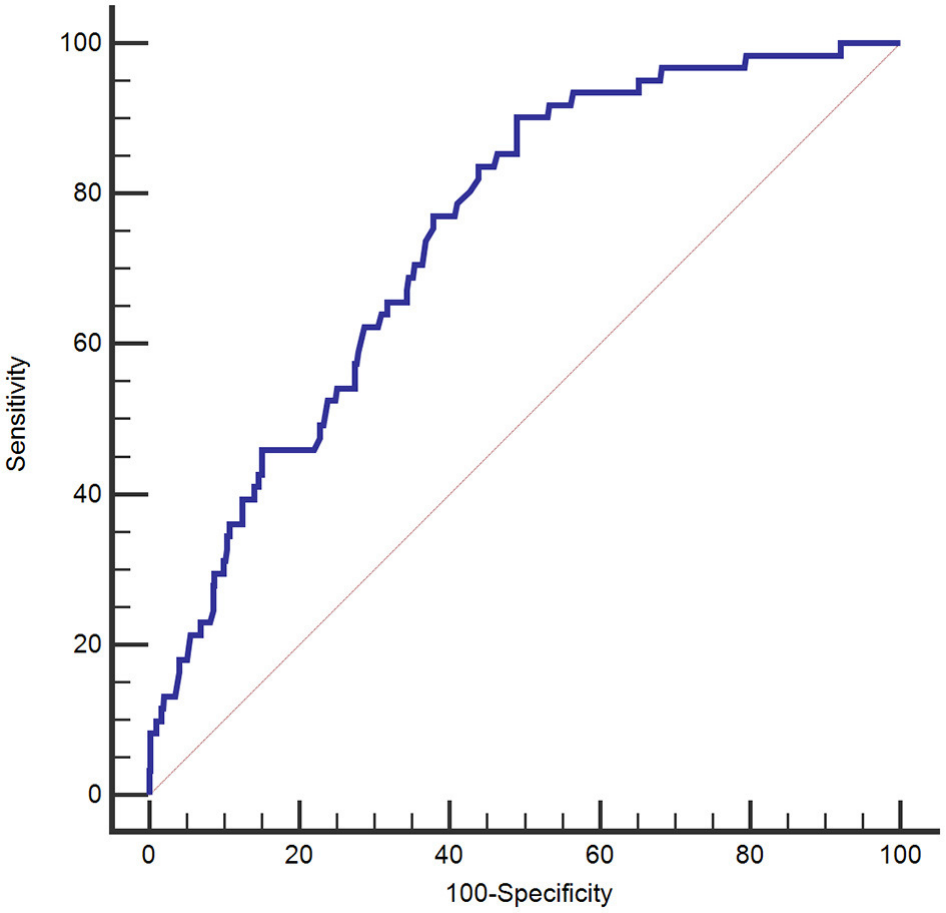
Receiver operating characteristic curve of the plasma glucose to potassium ratio for 3-month mortality of patients with aneurysmal subarachnoid hemorrhage.

Regarding the ROC curve for predicting 3-month mortality, the AUC for GPR was 0.747 (95% CI 0.709–0.783). The suitable cutoff of GPR for predicting mortality was determined to be 37.8 (sensitivity, 90.2%; specificity, 51.0%; negative likelihood ratio, 0.19; positive likelihood ratio, 1.84) (Figure 3).

Age, sex, HH score, modified Fisher score, GCS score, WBC count, hemoglobin level, D-dimer level, glucose level, potassium level, and GPR were included in the logistic regression analysis. In univariable analysis revealed that age, HH score, modified Fisher score, GCS score, hemoglobin level, glucose level, and GPR were significantly related to 3-month mortality. In multivariable analysis, independent predictors for 3-month mortality were as follows: GPR (OR 1.070, 95% CI 1.047–1.093; *p* < 0.001), and GCS score (OR 0.644, 95% CI 0.544–0.763; *p* < 0.001) (Table 4).

**Table 4:** Variables associated with 3-month mortality identified by logistic regression analysis.

	**Univariable**			**Multiple**		
	**Odds ratio**	**95% CI**	***P*-value**	**Odds ratio**	**95% CI**	***P*-value**
Age	1.043	1.021–1.065	<0.001	1.026	0.970–1.085	0.364
Sex	0.994	0.580–1.703	0.983			
Time from onset to ED admission	0.997	0.994–1.001	0.103			
Hunt and Hess score	6.153	3.966–9.546	<0.001	1.655	0.360–7.616	0.518
Modified Fisher score	5.782	3.122–10.711	<0.001	1.291	0.370–4.503	0.688
GCS score	0.632	0.573–0.699	<0.001	0.644	0.544–0.763	<0.001
Glucose	1.021	1.014–1.027	<0.001	1.033	0.995–1.072	0.094
Potassium	0.652	0.362–1.173	0.153			
Glucose/potassium ratio	1.052	1.035–1.069	<0.001	1.070	1.047–1.093	<0.001
WBC	1.000	1.000–1.011	0.652			
Hemoglobin	0.802	0.659–0.975	0.027	0.820	0.584–1.236	0.395
D-dimer	0.715	0.428–1.194	0.200			

*GCS, Glasgow Coma Scale; WBC, white blood cell*.

We evaluated the incremental benefit of adding the GCS score to the GPR by calculating the AUC; adding the GCS score to the GPR improved the predictive value for 3-month mortality (AUC = 0.925, 95% CI 0.888–0.963; *p* < 0.001).

### Discussion

This study investigated the association of plasma GPR with mortality among patients with aSAH. The plasma GPR on ED admission was significantly higher in non-survivors and was an independent predictor for 3-month mortality. Therefore, the plasma GPR was useful for predicting mortality in these aSAH patients.

Many factors stimulate the sympathetic nervous system in aSAH patients. Sympathetic activation leads to an increased release of catecholamine and cortisol into the systemic circulation, which begins a few minutes after an aSAH and can last for up to 10 days. These hormones stimulate gluconeogenesis, glycogenolysis, lipolysis, and proteolysis, which lead to glucose overproduction (10).

Previous studies have suggested that hyperglycemia is related with poor outcome in aSAH patients. Beseoglu and Steiger reported that hyperglycemia correlated with the initial neurological status and unfavorable outcome after 6 months (11). In the study by McGirt et al., patients with persistent high blood glucose levels were seven-fold more likely to have a poor prognosis than patients with good glucose control, although an isolated hyperglycemic event was not predictor for poor outcome (12). In our study, the plasma glucose level *at ED admission* was significantly higher in patients with a poor outcome and non-survivors; however, it was not an independent predictor for 3-month mortality. Unfortunately, our study did not evaluate the effect of glucose management on the prognosis of patients with aSAH during hospitalization.

A previous study reported that the difference in the risk of unfavorable outcomes between patients in the lowest and highest quartiles of glucose level was more than double (13). We found a 2.8-fold difference in the risk of poor outcomes and a 14.7-fold difference in mortality between the lowest GPR (<32.7) and the highest GPR (>47.2) quartiles.

Various factors such as systemic inflammatory response syndrome, sepsis, metabolic crisis, and insulin treatment may influence glucose control in patients with SAH (14). A previous study found that the infection rate was significantly lower in patients who were treated with intensive insulin therapy than it was in those without such treatment, but neurological outcome and mortality were not affected by intensive insulin therapy (15). Implementation of strict glucose control lowered the average glucose levels and incidence of hyperglycemia in patients compared with those without such control, but had no effect on hospital mortality (16). By contrast, good glucose management significantly reduced the incidence of poor outcome compared with that in patients with poor glucose management (17). Whether, control of hyperglycemia improves outcomes, including hypoglycemia, which can occur due to the control of hyperglycemia, or increases mortality remains unclear. Therefore, control of blood glucose levels above 180 mg/dl is currently recommended (18).

Hypokalemia is common electrolyte imbalance in aSAH patients. It is believed that hypokalemia associated with aSAH is induced by a catecholamine surge. A high catecholamine level causes overactivation of the Na^+^/K^+^-ATPase pump, which causes a shift in potassium ions from extracellular to intracellular spaces (19). Previous research found that about 50% of SAH patients had hypokalemia (20). In the study by Zhang et al., the prevalence of hypokalemia among aSAH patients was 35% (21). In our study, 26% of patients had hypokalemia, but this rate did not differ significantly between survivor and non-survivors. Only 20 patients in our study had moderate hypokalemia and no patients with severe hypokalemia. Tam et al. found that neither hypokalemia nor hyperkalemia were independent predictors for poor outcome at 3 months after SAH onset (22). By contrast, others found that only 2% of the patients included were hypokalemic on ED admission, and hypokalemia in the subacute phase (days 7–10) correlated with poor outcome at 3 months after discharge (23). The effect of a low potassium level on the outcome in aSAH patients remains controversial.

Recently, Fujiki et al. reported that serum GPR, glucose and potassium level at admission were significantly correlated with poor outcome at 3 months (7). Our results do not entirely coincide with their results. Interestingly, our study showed that plasma glucose was significantly higher in non-survivors than in survivors, but it was not an independent predictor of 3-month mortality in aSAH patients. These differences in results may be due to differences in study protocol and enrolled criteria, particularly the different severity of patients with aSAH. In the study by Fujiki et al., patients with various onset times from 1 h to 16 days were included, and 42.1% patients were classified as severe aSAH. On the other hand, our study included only aSAH patients whose onset time was within 24 h, and the proportion of severe patients was 24.2%.

The pathophysiological mechanism for the relationship between high plasma GPR and mortality in aSAH patients remains unclear. Hyperglycemia after SAH is associated with cardiac dysfunction, pulmonary edema, brain stem compression, bloodstream infection, and an increased risk of death (24). Furthermore, hyperglycemia induced intracellular acidosis, mitochondrial dysfunction, formation of free radicals, and brain edema (25). Severe hypokalemia increases the risk of cardiac arrhythmias, especially tachyarrhythmias. High GPR has been shown to be associated with the severity of SAH (7). In accordance with previous findings, our data also demonstrated that plasma GPR was significantly elevated in patients with severe aSAH compared with patients with non-severe aSAH. Whether, hyperglycemia and hypokalemia are factors that adversely affect the outcome of patients with aSAH or are simply surrogate markers of underlying etiological factors for poor outcome in clinical course remains controversial.

However, the plasma GPR was significantly elevated in non-survival patients and was an independent predictor for 3-month mortality. The relationship between 3-month mortality and glucose level on ED admission was weaker than that of the GPR and was no longer significant after multiple logistic regression analysis.

In our study, the AUC of the plasma GPR ranged from 0.709 to 0.783. For a cutoff value of 37.8 for the GPR, the sensitivity and specificity for predicting 3-month mortality were 90.2 and 51.0%, respectively. The Kaplan–Meier survival curves for 3-month mortality showed that patients in the group with the highest GPR (>47.2) had a significantly shorter overall survival time than those in the group with the lowest GPR (<32.7).

Our study has several limitations. First, glucose level can be influenced by catecholamine, cortisol, and glucagon levels, but we did not measure these. Second, glucose and potassium levels may be influenced by various factors such as a timing of last meal, use of beta-blockers, or potassium wasting diuretics, but we did not consider these factors. In addition, the effects of the management of glucose and potassium during hospitalization were not considered. Third, the plasma GPR was calculated only once based on the initial values obtained on admission to the ED, and it remains unclear whether serial measurement of GPR may provide additional prognostic information. In addition, the present data represents only a single tertiary hospital, and no *post-hoc* analysis was performed. Therefore, the outcomes cannot be generalized to all aSAH patients. Further prospective large-scale studies are required to confirm the relationship between GPR and mortality in aSAH patients.

In conclusion, plasma glucose and potassium level tests are inexpensive measures that are readily available from the routine blood analysis obtained at the time of admission to the ED. The plasma GPR was significantly higher in aSAH non-survivor than in survivor and has potential to be a prognostic predictor of 3-month mortality in aSAH patients.

## Data Availability Statement

The raw data supporting the conclusions of this article will be made available by the authors, without undue reservation.

## Ethics Statement

The studies involving human participants were reviewed and approved by Konkuk University Medical Center. Written informed consent for participation was not required for this study in accordance with the national legislation and the institutional requirements. Written informed consent was not obtained from the individual(s) for the publication of any potentially identifiable images or data included in this article.

## Author Contributions

DH and HJ conceived and designed the study. DH, JP, and SK collected and complied data. DH, HJ, JP, and SK performed statistical analysis and interpreted the data. HJ and DH wrote the report. All authors contributed to the article and approved the submitted version.

## Conflict of Interest

The authors declare that the research was conducted in the absence of any commercial or financial relationships that could be construed as a potential conflict of interest.
